# A clinically relevant method to screen for hepatic steatosis in overweight adolescents: a cross sectional study

**DOI:** 10.1186/s12887-015-0465-x

**Published:** 2015-10-08

**Authors:** Vera Saad, Brandy Wicklow, Kristy Wittmeier, Jacqueline Hay, Andrea MacIntosh, Niranjan Venugopal, Lawrence Ryner, Lori Berard, Jonathan McGavock

**Affiliations:** Children’s Hospital Research Institute of Manitoba, 511 JBRC. 715 McDermot Avenue, Winnipeg, Mb R3E 3P4 Canada; Department of Pediatrics and Child Health, Faculty of Health Sciences, College of Medicine University of Manitoba, Manitoba Institute of Child Health, 511 JBRC. 715 McDermot Avenue, Winnipeg, MB R3E 3P4 Canada; George and Fay Yee Centre for Healthcare Innovation, 300 Chown Building, 753 McDermot Avenue, Winnipeg, MB R3E 0T6 Canada; CancerCare Manitoba, 675 McDermot Avenue, Winnipeg, MB R3E 0 V9 Canada; The Diabetes Research Group, Department of Internal Medicine, Faculty of Medicine, University of Manitoba, 835 McDermot Avenue, Winnipeg, MB R3E 0 T8 Canada; Diabetes Research Envisioned and Accomplished in Manitoba Theme, 715 McDermot Avenue, Winnipeg, Mb R3E 3P4 Canada

**Keywords:** Fatty liver, ALT, Magnetic resonance spectroscopy, Adolescents, Obesity, Lipotoxicity

## Abstract

**Background:**

To develop a screening algorithm to detect hepatic steatosis in overweight and obese adolescents.

**Methods:**

We performed a cross sectional study of 129 overweight adolescents 13–18 yrs. The primary outcome, hepatic steatosis was defined as an intracellular triglyceride content > 5.5 mg/g and quantified using ^1^H-magenetic resonance spectroscopy. Primary predictor variables included, alanine and aspartate transaminases (ALT/AST) and features of the metabolic syndrome.

**Results:**

Hepatic steatosis was present in 33 % of overweight and obese adolescents. Adolescents with hepatic steatosis were more likely to be boys (adjusted OR: 4.8; 95 % CI: 2.5–10.5), display a higher waist circumference (111 ± 12 vs 100 ± 13 cm, *p* < 0.001) and have metabolic syndrome (adjusted OR: 5.1; 95 % CI: 1.6–16.4). Serum ALT predicted hepatic steatosis in boys (AUC: 0.82; 95 % CI: 0.70–0.95; *p* < 0.001) but not girls (AUC = 0.63; 95 % CI: 0.46–0.75, *p* = 0.16). An ALT >20 U/L, combined with the presence of metabolic syndrome, male gender and an elevated waist circumference provided the best model (AUC 0.85) with high sensitivity (72 %) and specificity (82 %) and positive and negative predictive values of 61 % and 89 % respectively.

**Conclusions:**

Serum transaminases provide modest predictive value for hepatic steatosis in youth. The ALT threshold for predicting hepatic steatosis is significantly lower than current clinical thresholds for predicting non-alcoholic fatty liver disease. The addition of ALT, presence of the metabolic syndrome and male gender significant improve the ability to predict hepatic steatosis.

## Introduction

Non-alcoholic fatty liver disease (NAFLD) is the most common cause of liver disease in children [1]. The prevalence of NAFLD has increased in parallel with the rise in childhood obesity [2]. NAFLD is a spectrum term that includes several stages of liver disease including the earliest stage of simple hepatic steatosis, the more severe non-alcoholic steatohepatitis and precedes the advanced stage of cirrhosis [3]. Population- and clinic-based studies suggest that 25–47 % of overweight and obese youth display some form of liver disease along the spectrum of NAFLD [2, 4, 5]. The clinical diagnosis of NAFLD relies initially on the detection of elevated serum transaminases followed by confirmation with hepatic ultrasound and finally a liver biopsy to score the degree of cellular damage, inflammation and fatty infiltration [3, 6, 7]. Current guidelines recommend the use of alanine aminotransferase (ALT) to initially screen for NAFLD in obese youth within community practice settings [8]. However, the appropriate ALT value for detecting hepatic steatosis, the earliest stage in the natural history of NAFLD, is unknown and current clinical thresholds significantly exceed the upper limit of normal for metabolically healthy youth [9].

The lack of consensus regarding the appropriate serum transaminase thresholds to detect hepatic steatosis is related, in part, to the scarcity of studies that measure hepatic triglyceride directly. The Screening ALT for Elevation in Today’s Youth (SAFETY) study recently reported that among otherwise healthy children, the 95^th^ percentile for ALT is ~26 U/L for boys and ~23U/L for girls, suggesting much lower ALT thresholds should be used to initially screen for chronic liver disease in children [9]. Thresholds based on the 95^th^ percentile provided high sensitivity and specificity for the detection of biopsy-proven NAFLD in obese adolescents [9]. Unfortunately, the study failed to identify serum transaminase thresholds for adolescents with hepatic steatosis alone, prior to the progression to the more extreme non-alcoholic steatohepatitis (NASH) [4, 10]. Identifying overweight youth in the earliest stages of fatty liver disease may be important for the early detection and prevention of progression to NAFLD or other associated metabolic disorders [4, 10–12].

In an effort to overcome these limitations, we performed a cross sectional study of [1] H-magnetic resonance spectroscopy-derived measures of hepatic triglyceride content and serum measures of liver transaminases in a sample of overweight and obese adolescents enrolled in a therapeutic trial to reduce hepatic triglyceride content. We hypothesised that the ALT thresholds with the best sensitivity and specificity for detecting hepatic steatosis in overweight and obese youth would be lower than current clinical cut-points. A secondary aim was to develop an algorithm using commonly measured metabolic risk factors to predict hepatic steatosis that would be useful in a community pediatric outpatient setting.

## Research design and methods

### Study design and study population

Between 2008 and 2012, 181 youth aged 13–19 years were screened for participation in a randomized controlled trial of physical activity on risk factors associated with the development type 2 diabetes (www.clinicaltrials.gov; NCT00755547). Of the 181 adolescents screened, 129 were overweight or obese and provided valid measures of hepatic triglyceride content as well as measures of serum liver transaminases, cholesterol, blood pressure and waist circumference and were included in this cross sectional study [4, 10]. Participants were classified as overweight or obese according to age- and sex-specific BMI cut points established by the International Obesity Task Force [13]. All participants were screened with a 75 g 2-hr oral glucose tolerance test and those with a diagnosis of type 2 diabetes or impaired glucose tolerance were excluded. We also excluded adolescents (1) treated with antipsychotics, hepatotoxic medications or corticosteroids, (2) with infectious causes of hepatitis, (3) reporting frequent binge drinking; (4) other self-reported concomitant liver diseases or (5) enrolment in a weight loss program in the 6 months prior to their first study visit. Adolescents who were unable to undergo MRI due to weight or size restrictions were also excluded. Among the 52 adolescents that failed to meet inclusion criteria, 29 were excluded because of impaired glucose tolerance or type 2 diabetes during the initial screening phase, 8 did not have a measure of hepatic triglyceride content and 15 were not overweight or obese. All participants and parents provided written informed consent for observational studies of tissue steatosis and insulin resistance in youth as well as participation in the randomized controlled trial (NCT00755547). The study was approved by the University of Manitoba Biomedical Research Ethics Board (B2006:091) and the National Research Council of Canada (W2007-04) in accordance with the Declaration of Helsinki.

### Primary outcome measure: hepatic steatosis

Hepatic triglyceride content was quantified using magnetic resonance spectroscopy on a 1.5 or 3.0 T whole body magnet (GE Medical Systems, Milwaukee, WI) [4, 10, 14, 15]. Using MRI-derived high resolution images, a single voxel (40 mm^3^) was prescribed within the upper right lobe of the liver in an area devoid of subcutaneous or visceral fat as to prevent unwanted lipid contamination from peripheral tissue. To further prevent peripheral lipid contamination, several spatial saturation bands which act to null peripheral lipid signals were manually placed around the voxel. Using the PRESS based localization sequence, with TE = 25 ms and TR = 3 s, we acquired a total of 64 spectra and 1,024 data points over a 1,000-Hz spectral width. LCModel software was used to isolate and quantify lipid and water peaks [4, 10, 16]. Hepatic steatosis was defined as hepatic triglyceride content of >5.5 % fat/water based on previous population-based studies and the observation that it is equivalent to a biopsy-derived lipid concentration of 5.5 mg/g [3, 15].

### Predictor variables

Serum alanine (ALT) and aspartate transaminase levels (AST) were treated as continuous variables and measured on a Roche Modular P Analyze after a 10-hr overnight fast. Metabolic syndrome was treated as a binary outcome measure using cut points for systolic blood pressure, serum triglycerides, waist circumference, fasting glucose, and HDL- cholesterol (HDL-C) that were statistically derived to reflect cut points in adults [17]. Adolescents were described as having metabolic syndrome if they had three or more of five comorbidities [17]. Resting systolic and diastolic blood pressure were measured in triplicate in a sitting position using a Dinamap automatic machine, as recommended by the National Committee on Preventive, Detection, Evaluation and Treatment of High Blood Pressure [18]. Plasma glucose was measured on a Roche Modular P analyzer using the hexokinase method. LDL cholesterol (LDL-C) was calculated using the Friedewald equation (LDL-C = total cholesterol − [HDL-C − (triglyceride/2.2)]) [10]. Insulin was measured on an Immulite chemiluminescent immunometric assay. HOMA-IR was calculated using a standard formula [19]. Ethnicity was self-reported by parents and/or adolescents.

Body weight was measured to the nearest 0.1 kg on a calibrated scale. Height was obtained with a standard stadiometer and measured to the nearest 0.5 cm. Absolute body mass index (kg/m2) was converted to a BMI Z-score using nationally representative age and sex specific normative data using EpiInfo software [20]. Dual-energy X-ray absorptiometry (Hologic, Bedford, MA) was used to quantify percent body fat, total fat mass and fat free mass.

### Statistical analysis

Descriptive data are presented as mean ± SD or proportions where appropriate. Differences in demographic variables between youth with and without hepatic steatosis were performed using independent T-tests or Mann Whitney U tests where appropriate. The primary outcome for all regression analyses and receiver operating curves was hepatic steatosis, treated as a binary outcome (>5.5 % fat/water). Univariate analyses between predictor variables and hepatic steatosis were performed using Kruskal-Wallis and chi-square analyses as appropriate. Area under the curve (AUC), sensitivity, specificity, and positive- and negative-predictive values for the use of ALT for predicting hepatic steatosis were calculated from univariate logistic regression models, both for the combined sample and separately by gender. Youden’s J Statistic was used to determine the optimum cut-off value for ALT to predict the presence of hepatic steatosis. Based on the results of a univariate analysis, six variables were identified and entered into a multiple linear regression for predicting hepatic steatosis. BMI Z Score, ethnicity, metabolic syndrome, sex, waist circumference and ALT were entered into the model to estimate AUC as well as parameter estimates. A second multivariate model with ALT dichotomized at 20 was fit as it was determined to be the optimal cut-point to predict hepatic steatosis, based on the results of unadjusted receiver operating curves for predicting hepatic steatosis. Non-significant parameters were then removed, producing the final model. All analyses were performed with SAS Version 9.3 (SAS Institute, Cary NC).

## Results

Participant demographics stratified according to the presence of hepatic steatosis are provided in Table 1. Among the 129 youth studied, 33 % (*n* = 42) displayed hepatic steatosis. Compared to youth without hepatic steatosis, those with steatosis were more likely to be boys (OR: 4.8; 95 % CI: 2.5–10.5, *p* < 0.001), displayed a higher BMI Z score, higher waist circumference and were more likely to have the metabolic syndrome (OR: 6.7; 95 % CI: 3.0–15.2; *p* < 0.001). Youth with hepatic steatosis displayed ALT values nearly 2-fold higher than those without steatosis (*p* < 0.001), while AST values were only marginally higher (26 vs 21 U/L, p = 0.02).

**Table 1:** Participant characteristics stratified by the presence of hepatic steatosis

	Hepatic Steatosis (*n* = 43)	No Hepatic Steatosis (*n* = 82)	*P*-value
Female	24	64	<0.001
Age (yrs)	15 ± 2	15 ± 2	0.53
BMI (kg/m^2^)	33.8 ± 4.9	31 ± 4.5	0.002
BMI Z score	2.2 ± 0.4	1.9 ± 0.4	<0.001
Waist circumference (cm)	111 ± 12	100 ± 13	<0.001
Body fat percent (%)	38.9 ± 6.1	38 ± 5.9	0.43
Hepatic Triglyceride (%F/W)	13.0 ± 12.5	3.0 ± 1.2	<0.001
HOMA	6.4 ± 8.0	3.6 ± 3.2	0.43
HDL-cholesterol (mmol/L)	1.1 ± 0.3	1.2 ± 0.3	0.23
Triglycerides (mmol/L)	1.6 ± 0.7	1.1 ± 0.5	<0.001
AST (U/L)	26.4 ± 13.7	21.7 ± 8.8	0.008
ALT (U/L)	31.8 ± 22.8	19.0 ± 13.7	<0.001
Systolic BP (mmHg)	116 ± 12	114 ± 11	0.35
Diastolic BP (mmHg)	62 ± 8	65 ± 8	0.08
Metabolic syndrome	40 %	12 %	<0.001

Data are means ± standard deviation unless otherwise stated. *BMI* Body mass index, *HOMA-IR* homeostatic model assessment- insulin resistance, *HDL* High density lipoprotein, *AST* aspartate aminotransferase, *ALT* alanine aminotransferase, *BP* blood pressure

**Fig. 1:**
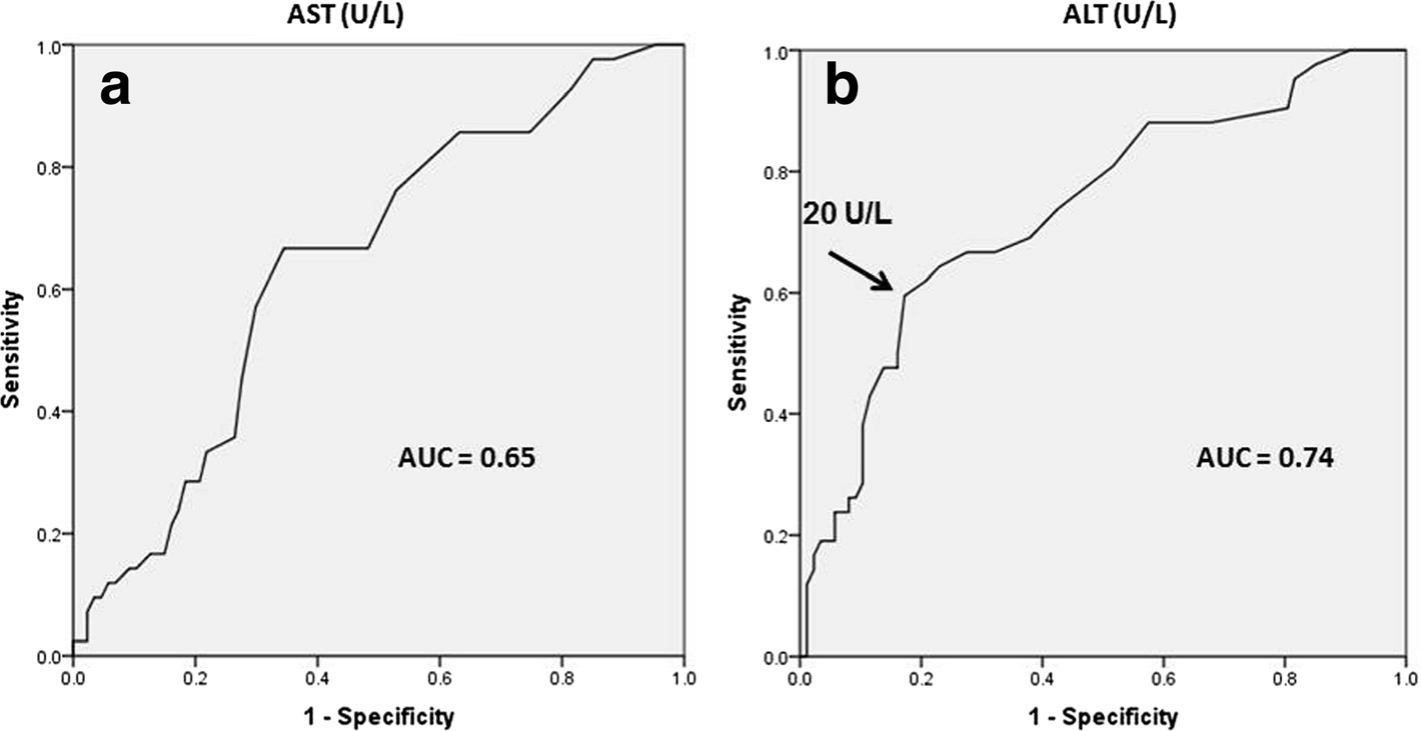
Receiver operating characteristic curves for predicting hepatic steatosis with serum transaminase values. **a** = Aspartate transaminase (*AST*); **b** = Alanine transaminase (*ALT*)

BMI Z score (AUC = 0.70; 95 % CI: 0.64–0.82, p–0.008) and waist circumference (AUC = 0.73; 95 % CI: 0.61–0.80; *p* < 0.001) were both modest but significant predictors of hepatic steatosis. AST (AUC: 0.65; 95 % CI: 0.55–0.74, *p* = 0.008) provided poor predictive value for the presence of hepatic steatosis (Fig. 1a). ALT (AUC: 0.74; 95 % CI: 0.64–0.83, *p* < 0.001) provided modest predictive value for the presence of hepatic steatosis (Fig. 1b), with an area under the curve similar to that provided by BMI Z score and waist circumference. Sensitivity and specificity for various ALT thresholds with corresponding positive and negative predictive values are presented in Table 2a. An ALT level of 20 U/L provided the highest acceptable sensitivity (64.3 %) with the lowest acceptable compromise in specificity (74.7 %). While higher thresholds of ALT provided superior specificity, rates of false negatives increased ~2-fold (18–30 %) and the negative predictive value decreased from 82–71 % (Table 2). As rates of hepatic steatosis were significantly higher among boys, we also provided similar data across a range of ALT values for boys alone (Table 2b). Due to the low rates of steatosis in girls, a similar table was not possible to generate.

**Table 2:** a ALT threshold levels used in screening for hepatic steatosis and corresponding sensitivities and specificities for all participants

ALT (U/L)	Sensitivity	Specificity	Positive predictive value	Negative predictive value
>20	64 %	77 %	57 %	82 %
>25	48 %	86 %	62 %	77 %
>30	33 %	90 %	61 %	74 %
>35	24 %	92 %	59 %	71 %
>40	19 %	94 %	62 %	71 %
>66 U/L	10 %	99 %	75 %	71 %
b				
ALT threshold levels used in screening for hepatic steatosis and corresponding sensitivities and specificities for boys only				
ALT (U/L)	Sensitivity	Specificity	Positive predictive value	Negative predictive value
>20	79 %	63 %	73 %	71 %
>25	66 %	78 %	80 %	65 %
>30	45 %	89 %	85 %	57 %
>35	38 %	89 %	82 %	53 %
>40	29 %	95 %	88 %	51 %
>66 U/L	20 %	100 %	100 %	50 %

**Fig. 2:**
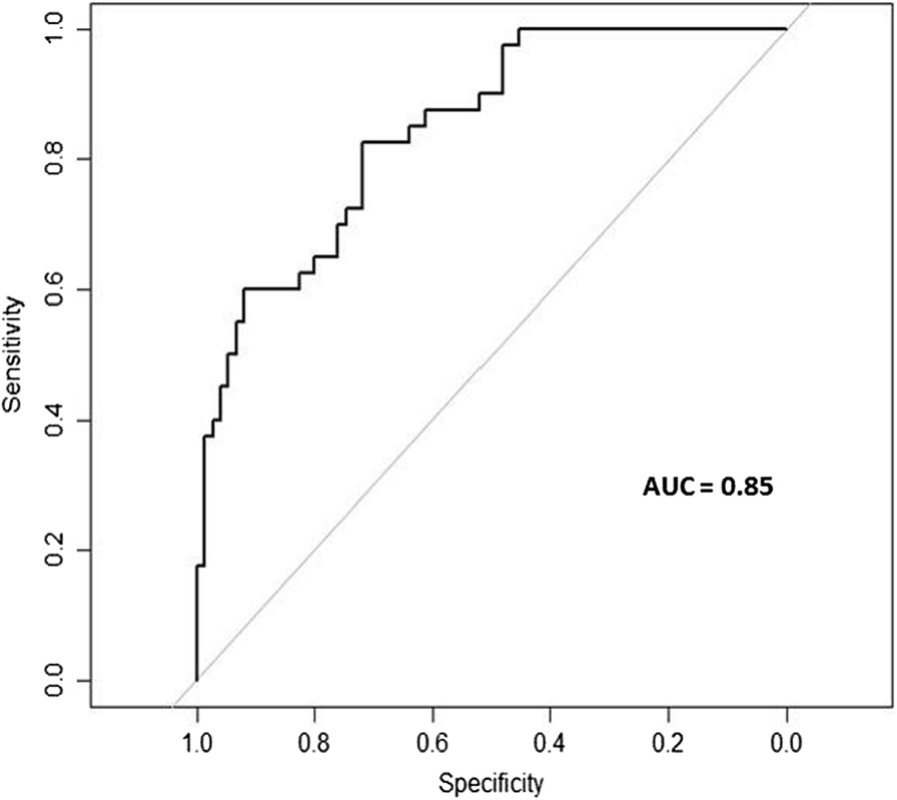
Receiver operating curve for the utility of the new algorithm for predicting the presence of hepatic steatosis in overweight and obese adolescents Results of a multiple regression analysis that included ALT > 20 U/L, male sex, waist circumference and the presence of the metabolic syndrome

While ethnicity, and BMI Z score were associated with hepatic steatosis in univariate models, they were not significantly associated with hepatic steatosis among overweight youth in the multivariate logistic regression models (Table 3). The final multivariate logistic model included the presence of metabolic syndrome (vs no metabolic syndrome; aOR: 5.1; 95 % CI: 1.6–16.4); male sex (aOR: 5.5; 95 % CI: 1.9–16.2), an ALT > 20 U/L (aOR: 3.1; 95 % CI: 1.5–9.4) and waist circumference (aOR: 1.06; 95 % CI: 1.02–1.10) (Table 4). The presence of one or two individual components of the metabolic syndrome were not associated with hepatic steatosis in this cohort, suggesting that the presence of a minimum of three risk factors is needed to predict of hepatic steatosis in overweight/obese adolescents. Receiver operating characteristic curves that combined all four criteria yielded an AUC of 0.85 (*p* = 0.001) (Fig. 2b) with high levels of sensitivity (0.72) and specificity (0.82).

**Table 3:** Predictors of hepatic steatosis in overweight and obese adolescents

	Point Estimate	95 % CI
BMI Z score	2.67	0.94–9.67
Indigenous vs Other	0.59	0.12–2.96
Caucasian vs Other	0.75	0.17–3.27
Metabolic Syndrome		
Components		
1 vs 0	0.95	0.14–6.50
2 vs 0	1.64	0.26–10.52
3 or 4 vs 0	6.54	0.87–49.05
Sex (M vs F)	3.61	1.31–9.93
AST (U/L)	3.14	1.22–8.09

**Table 4:** Conventional variables used to predict hepatic steatosis in obese adolescents

	Point Estimate	95 % CI
MS 1 vs 0	1.05	0.16–7.05
MS 2 vs 0	2.09	0.33–13.26
MS 3 or 4 vs 0	8.34	1.16–60.0
Sex (M vs F)	4.40	1.64–11.83
AST (U/L) > 19	3.74	1.49–9.38

*MS* metabolic syndrome count, *AST* aspartate transaminase

A significant interaction between ALT and sex was noted in preliminary analyses, therefore regression models were constructed for boys and girls separately. Among boys, the combination of presence of the metabolic syndrome and ALT > 20 U/L provided an area under the curve of 0.90 (95 % CI: 0.82–0.99). Among girls, the presence of the metabolic syndrome and an ALT > 20 U/L yielded an area under the curve of 0.74 (95 % CI: 0.58–0.89).

## Discussion

To the best of our knowledge, this is the first diagnostic study designed to identify a threshold for liver transaminases that predicts objectively measured hepatic steatosis using magnetic resonance spectroscopy in overweight/obese adolescents. The data build on the extensive work of Nobili and colleagues [1, 21–24] and others [9], by demonstrating that the threshold for ALT with the best balance of positive and negative predictive value is much lower than current clinical standards. Furthermore, the findings presented here extend previous studies of overweight and obese youth [9, 23], by providing a novel algorithm that detects hepatic steatosis.

Hepatic steatosis is one of the most common complications of obesity in children and adolescents [1, 3] and a challenge for general pediatricians to detect and treat [21]. Within the pediatric clinical settings, liver transaminases are used to initially screen for the presence of fatty liver disease [10, 18], however recent studies suggest a simple measure of transaminases in not sufficient to detect hepatic steatosis or NAFLD [25]. Non-invasive imaging tools, such as magnetic imaging and ultrasound, provide semi-quantitative insight into the degree of steatosis [26–28], however are not generally used in general pediatric settings. The threshold at which youth are considered “at risk” of NAFLD varies widely across settings (30 – 66 U/L or “two-fold higher than normal”) due in large part to the reliance on local measures of the upper limits of normal [1, 9]. The SAFETY study recently determined that (1) cut-off values are set too high for reliable detection of pediatric chronic liver disease (In fact, hepatic steatosis was detectable at ALT values 10–50 % lower than conventional thresholds (25 U/L vs 30–66 U/L)) and (2) that these lower cut points are sensitive and specific to detecting liver diseases, including NAFLD, in children and adolescents [9]. The data presented here support the notion that the current clinical thresholds are too high for detecting magnetic resonance spectroscopy-derived hepatic steatosis as the number of false negatives was ~2-fold higher using commonly used threshold (30 vs 18 %), compared to the lower threshold identified here. The data also reinforce the limited utility of ALT alone as a screening tool for hepatic steatosis in overweight/obese adolescents, as the area under the curve was similar to that for measures of adiposity. These data support the call from others that the thresholds for detecting fatty liver disease in children and adolescents need to be revised and harmonized across pediatric clinical settings.

The metabolic syndrome consists of a clustering of cardiometabolic risk factors that, in adults, is associated with cardiovascular disease and type 2 diabetes [29, 30]. The metabolic syndrome in childhood is a strong predictor of impaired glucose tolerance and progression to type 2 diabetes in adulthood [31]. Our group and others have documented that hepatic steatosis is a robust predictor of metabolic syndrome and type 2 diabetes in overweight and obese adolescents [1, 4, 10, 22, 24, 30]. It is not surprising therefore, that adding the presence of 3 or more metabolic syndrome features to a measure of ALT provides significantly greater predictive power for detecting hepatic steatosis in overweight/obese adolescents. Importantly, the presence of one or two individual risk factors was not predictive of hepatic steatosis in adolescents, reinforcing the notion that hepatic steatosis and the metabolic syndrome are intimately linked. The presence of visceral obesity is likely an important mediator of this association as it is often associated with both conditions [32] and the observation that waist circumference was positively associated with hepatic steatosis in this study, reinforces it's utility in the clinical assessment of obese adolescents. From a clinical standpoint, these data reinforce the concept that cardiometabolic risk factors tend to cluster in overweight and obese youth, which may be a harbinger of clinically relevant cardiometabolic endpoints.

Sex differences exist in the partitioning of adipose tissue in adults and youth [33]. The deposition of adipose tissue in the visceral space is more common among boys and men [33] and is generally highly correlated with the presence of hepatic steatosis [34]. Biopsy studies support these observations, demonstrating that NAFLD is more common in overweight boys than girls [35]. The data presented here support these studies and population-based studies of hepatic steatosis using magnetic resonance spectroscopy [15, 36, 37] as the presence of hepatic steatosis was ~5-fold higher in boys compared to girls. Based on the sex-based differences in the presence of hepatic steatosis, the upper limits of normal for transaminase levels are general higher for boys, relative to girls [9, 14]. The ALT threshold we identified for the optimal detection of hepatic steatosis in the current study was very similar to the threshold used to detect biopsy-proven NAFLD among boys [9] (20 vs 27 U/L). Interestingly, the utility of ALT for predicting hepatic steatosis was poor among overweight and obese girls, relative to boys (AUC = 0.73 vs 0.90). This may reflect gender-specific consequences of lipotoxicity on hepatocytes, or different thresholds of intracellular triglyceride content at which liver enzymes are released. Large population-based studies and biopsy-based clinical investigations are needed to explore the mechanisms for sex differences in the risk of hepatic steatosis.

The current study expands on previous studies as we relied on magnetic resonance spectroscopy to quantify hepatic triglyceride content in a relatively large community-based sample of overweight and obese adolescents at a presumable early stage of NAFLD. The study is also strengthened by the use of predictor variables that are commonly used in both hospital and community-based pediatric care settings. Several limitations in the current study design however, need to be addressed. As hepatic biopsies were not performed on youth in this sample, it is impossible to rule out the presence of NAFLD in those with >5.5 % liver fat (i.e. hepatic steatosis) using single voxel MR spectroscopy, potentially skewing the thresholds for liver transaminases upwards. We feel this is unlikely to have significantly influenced our results as none of the adolescents self-reported a previous diagnosis of fatty liver disease or elevated liver enzymes prior to their study visit. Second, as this was a sample of youth recruited specifically for a randomized trial of exercise training, selected based on their risk for type 2 diabetes and the presence of low levels of self-reported physical activity, the study is at risk of selection bias and an overestimate of the prevalence of hepatic steatosis. Third, while the new algorithm for predicting the presence of hepatic steatosis is superior to using ALT alone, the sensitivity and specificity remain sub-optimal, therefore a diagnosis of hepatic steatosis should include imaging of the liver. Finally, as the study was cross sectional, and lacked a validation cohort, these findings need to be replicated and the time course of changes in hepatic triglyceride content and the increase in serum transaminase levels remains should be studied. Despite these limitations, the data presented here provide important initial insight into clinically-relevant predictors of hepatic steatosis in overweight and obese youth.

## Conclusions

The clinical thresholds for serum transaminases for detecting hepatic steatosis in overweight and obese youth is lower than the current recommended thresholds for identifying hepatic steatosis. The predictive value of ALT for detecting hepatic steatosis is significantly greater among overweight boys, than overweight girls. Finally, it is possible to predict the degree of hepatic steatosis with high sensitivity using a serum measure of ALT, sex, waist circumference and the presence of the metabolic syndrome.

## Abbreviations

AST: Asparate aminotransferase
ALT: Alanine aminotransferase
NAFLD: Non alcoholic fatty liver disease
